# Implementation of a COVID-19 Vaccine Emergency Department Education Program for Underserved Communities

**DOI:** 10.7759/cureus.30972

**Published:** 2022-11-01

**Authors:** Austin Schoeffler, Elizabeth J Bashian, Nathan Callender, Emily D Geyer, Aditya More, Tyler Webb, Jackiethia L Butsch, Nicholas E Kman, Jason J Bischof

**Affiliations:** 1 Emergency Medicine, The Ohio State University College of Medicine, Columbus, USA; 2 Community and Civic Engagement, The Ohio State University Wexner Medical Center, Columbus, USA; 3 Emergency Medicine, The Ohio State University Wexner Medical Center, Columbus, USA

**Keywords:** quality improvement, vaccine hesitancy, emergency department, vaccine education, covid-19

## Abstract

Background

The COVID-19 pandemic has provided an opportunity for significant reflection on our public health response as providers. Throughout the past two years, we learned that administration of COVID-19 vaccines, rapidly and widely across all communities, has been key to halting the spread of the virus. One significant challenge in promoting a large-scale immunization program is the threat of vaccine hesitancy. A general mistrust in healthcare providers exists across the country, especially in underrepresented minority (URM) communities.

Objective

This study aims to determine reasons for vaccine hesitancy in an urban emergency department and to provide targeted education on the safety and efficacy of the COVID-19 vaccines to patients.

Methods

An interprofessional quality improvement team was assembled to develop an educational intervention addressing COVID-19 vaccine safety for vaccine-eligible patients receiving treatment in the emergency department at an urban community hospital where over 70% of patients identify as URM. A survey was conducted to elucidate patients’ concerns surrounding the COVID-19 vaccine. Upon completion of the survey, up-to-date safety information and education targeting their surveyed concerns were provided by trained medical students. A follow-up survey was conducted to assess the impact of education on patients’ attitudes toward the vaccine. Surveys were developed using standardized scoring systems from the Oxford coronavirus explanations, attitudes, and narratives survey (OCEANS) II study and the Kaiser Foundation. Hesitancy scores before and after education were tabulated to assess the effectiveness of targeted education in improving vaccine hesitancy.

Results

Patients cited a variety of concerns surrounding the COVID-19 vaccine. The three most common reasons for declining vaccines were potential side effects (67.3% were concerned or extremely concerned), the belief that COVID-19 vaccines are neither effective nor safe (64.5% were concerned to extremely concerned), and the risk of developing COVID-19 infection from the vaccine itself (38.8% were concerned to extremely concerned). This information was used to address these concerns directly with patients, answer questions, clarify information, and encourage patients to get vaccinated. Through this education program, vaccine hesitancy scores improved by an average of 29% indicating an increased likelihood of patients who would get vaccinated in the future. Of patients receiving education, 38% agreed to sign up for a vaccine appointment during the intervention.

Conclusion

The emergency department often serves vulnerable patient populations. As such, its role in public health in these communities cannot be underestimated. This quality improvement project is a novel method that can be used to develop and implement public health education programs to address specific community needs in the emergency department. These results show that a multidisciplinary healthcare team can provide a measurable change in attitudes about vaccine safety with directed education in the emergency department that can help address vaccine hesitancy in the future.

## Introduction

The COVID-19 pandemic has been one of the greatest modern health challenges in history. With over 91 million infections throughout the United States in the past two years, this virus has taken a devastating toll on healthcare infrastructure and impacted every aspect of society. With the approval of several COVID-19 vaccines by the US Food and Drug Administration Emergency Use Authorization (EUA), the number of COVID-19 infections has declined dramatically in many locations around the United States. As of August 2022, over 67% of the United States population has been fully vaccinated with over 78% at least receiving one dose [1]. In Ohio specifically, over 63% of people have received at least one dose of vaccine [2]. While these numbers are promising, the road to achieving these milestones has been challenging.

One of the significant barriers to promoting a large-scale immunization program is vaccine hesitancy. It has been well documented that a general mistrust in healthcare providers exists in many communities across the country, especially in our underrepresented minority (URM) communities [3]. Disproportionate focus by the public on rare adverse events such as Guillain-Barre syndrome (GBS), myocarditis, thrombosis with thrombocytopenia syndrome (TTS), and anaphylaxis, combined with vaccine information on social media, led to concern in vaccine safety among many demographic groups [4].

The emergency department (ED) has a rapidly expanding scope of practice. It is no longer simply a place where acute injury and illness are treated but now serves as a location to engage in public health interventions as well [5]. Hundreds of patients are seen in emergency departments daily, with many of these patients lacking access to health information or a primary care provider with whom to discuss their medical concerns.

During the implementation of a large healthcare system’s vaccine program in early 2021, one of the major priorities was to make sure that URM communities were included in the rollout process. To improve vaccine uptake in this population, our goal was to gain a better understanding of the concerns of the community surrounding these vaccines. We planned to use this improved understanding of patients' concerns to develop and provide targeted education to those most at risk for declining the vaccine. In this project description, we detail an emergency department-based quality improvement project targeting COVID-19 vaccine education. This medical student-led project with support from ED physicians, social workers, and a community health worker aimed to combat vaccine hesitancy and increase vaccination rates in participants from URM communities seeking ED care in an academic affiliated, urban community hospital.

## Materials and methods

Overview

The objective of this quality improvement COVID-19 vaccine education program was twofold: 1) survey the current vaccine hesitancy landscape of an academic affiliated community hospital ED’s patient population, and 2) use this information to provide targeted education to at-risk patients about the COVID-19 vaccine and its potential side effects. To do this, a multidisciplinary group of physicians, medical students, social workers, and a community health worker came together to assess community needs.

Target population

The project was performed at Ohio State East Hospital, an academic affiliated, urban community hospital with an ED that sees more than 40,000 patient visits per year (26% white patients and 74% URM patients). This pilot project was implemented between January and July of 2021 with a convenience sample of 100 patients who were eligible to receive a COVID-19 vaccine but had not received a vaccine yet based on electronic medical record documentation. Exclusion criteria included patients with altered mental status unable to complete a survey or non-English language speakers. Of these 100 patients, 53 surveys were completed in their entirety.

Patient education process

**Figure 1:**
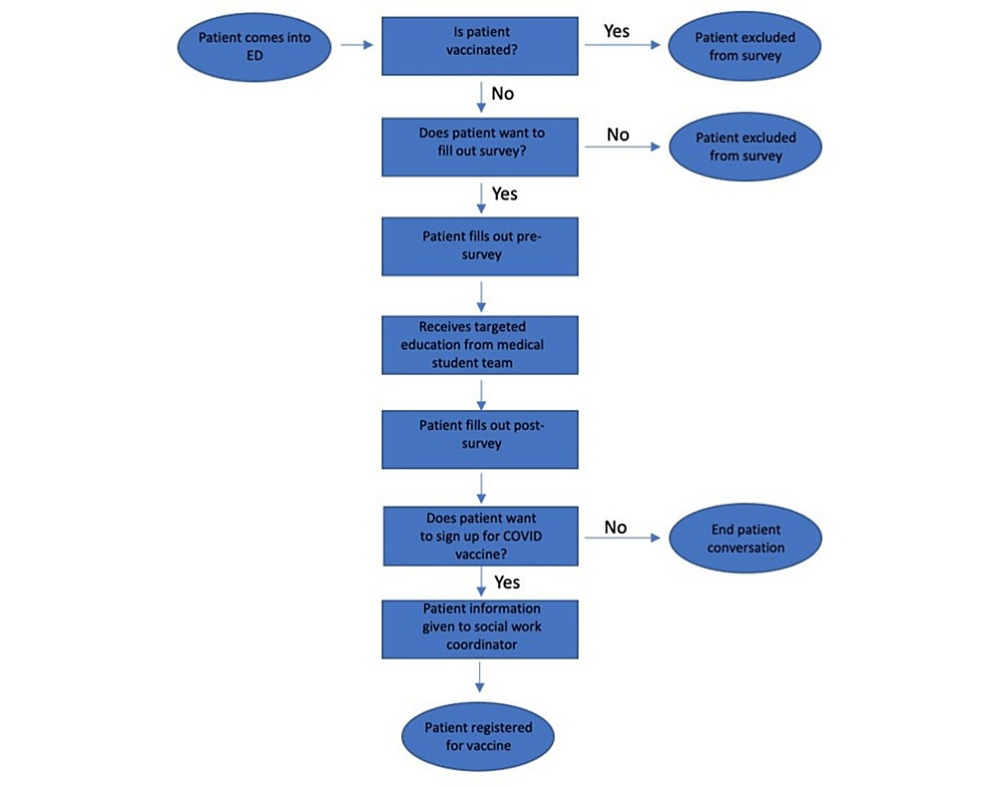
Process Map for Medical Student Education

As vaccines became available, the intervention team monitored the electronic medical record to screen for unvaccinated, eligible patients presenting to the ED. At the time of ED preparation for discharge, a medical student team member approached the patient and obtained verbal consent to participate in this quality improvement project. If patients agreed to participate, they were given an 11-question survey gauging the patient’s education level, vaccine hesitancy, and reasons that they might not have received the vaccine yet. Following this survey, a script (provided to all medical students) with high-yield information about the COVID-19 vaccination was discussed with each patient to standardize the education process. Based on the patient’s initial survey data, medical students would also directly address any additional patient questions surrounding COVID-19 vaccination. Medical students were provided with weekly information updates about the latest literature on COVID-19 vaccines to assure the accuracy of the education being provided. At the end of the interview, patients were given a handout with facts regarding COVID-19 vaccination and a step-by-step instruction guide on how to sign up for a vaccine. Lastly, a three-question follow-up survey was given to the patient to see if their vaccine hesitancy had improved. This protocol can be summarized in our process map (Figure 1).

Vaccine hesitancy survey

The survey was developed using a combination of data elements from the Kaiser Foundations COVID-19 Vaccine Monitoring Program [6] and the Oxford coronavirus explanations, attitudes, and narratives survey (OCEANS) II Vaccine Hesitancy Survey [7]. Three of these survey questions were provided both before and after patient education to gauge the effectiveness of our intervention in changing patient perceptions of the COVID-19 vaccine (Table 1). In collecting these responses before and after education, a patient's vaccine hesitancy score was calculated from 1 to 5 (1 meaning extremely hesitant, 5 meaning very likely to get the vaccine). These scores, as described in the OCEANS II study, were used to assess the level of impact on vaccine hesitancy. Six additional questions, derived from the Kaiser Foundations vaccine monitoring program, were provided during this survey to assess the most common reasons that patients might be hesitant to receive the COVID-19 vaccine (Table 2).

**Table 1:** Patients' Vaccine Hesitancy Score Questions

Question	Response
When an FDA-authorized vaccine for COVID-19 is available to you for free, do you think you will…?	Get it as soon as possible (5)
	Get it when I have time (4)
	Delay getting it (3)
	Avoid getting it for as long as possible (2)
	Never get it (1)
	Don’t know (-)
If my family or friends were thinking of getting a COVID-19 vaccine, I would…	Strongly encourage them (5)
	Encourage them (4)
	Not say anything to them about it (3)
	Ask them to delay getting the vaccine (2)
	Suggest that they do not get the vaccine (1)
	Don’t know (-)
Are you willing to sign up for an appointment to get the COVID-19 vaccine right now?	Yes
	No

**Table 2:** Patients' Vaccine Hesitancy Survey

How concerned are you about the following with the COVID-19 vaccine:	Response
Might experience serious side effects?	Extremity concerned (5)
	Very concerned (4)
	Concerned (3)
	Somewhat concerned (2)
	Not concerned at all (1)
Won’t be able to schedule and travel to vaccination site for the first/second dose?	Extremity concerned (5)
	Very concerned (4)
	Concerned (3)
	Somewhat concerned (2)
	Not concerned at all (1)
Might get COVID-19 from the vaccine?	Extremity concerned (5)
	Very concerned (4)
	Concerned (3)
	Somewhat concerned (2)
	Not concerned at all (1)
Might need to take time off work to get the vaccine/take off work if feeling sick after?	Extremity concerned (5)
	Very concerned (4)
	Concerned (3)
	Somewhat concerned (2)
	Not concerned at all (1)
The long-term effects of the COVID-19 vaccine are unknown	Extremity concerned (5)
	Very concerned (4)
	Concerned (3)
	Somewhat concerned (2)
	Not concerned at all (1)
The COVID-19 vaccines are not as safe/effective as they are said to be	Extremity concerned (5)
	Very concerned (4)
	Concerned (3)
	Somewhat concerned (2)
	Not concerned at all (1)

Social work/community health worker involvement

A combined team of community health and social workers were invaluable in reaching a large quantity of patients. With their assistance, discharge flags were set through the electronic medical record for patients who were eligible and had not received the vaccine yet. Our team was able to locate these individuals quickly as they were being discharged, discuss the vaccine, and assist in the vaccine registration process if required. Vaccine reminder cards were given to patients with the date, time, and location of their appointments. Additionally, our community health worker was able to follow up with patients who wanted to schedule appointments, set up a transportation system for patients with difficult access, and eliminate any barriers for our patients to get their vaccine. This process allowed for intervention without disrupting the workflow of their primary medical care in the ED.

Data storage and analysis

All information was stored and managed via REDCap (Vanderbilt University, Nashville, USA) electronic data capture tools hosted by our local institution [8]. Pearson’s χ^2^ was applied to determine if a significant difference (p < .05) existed between vaccine hesitancy scores before and after our education.

## Results

Vaccine hesitancy survey

**Figure 2:**
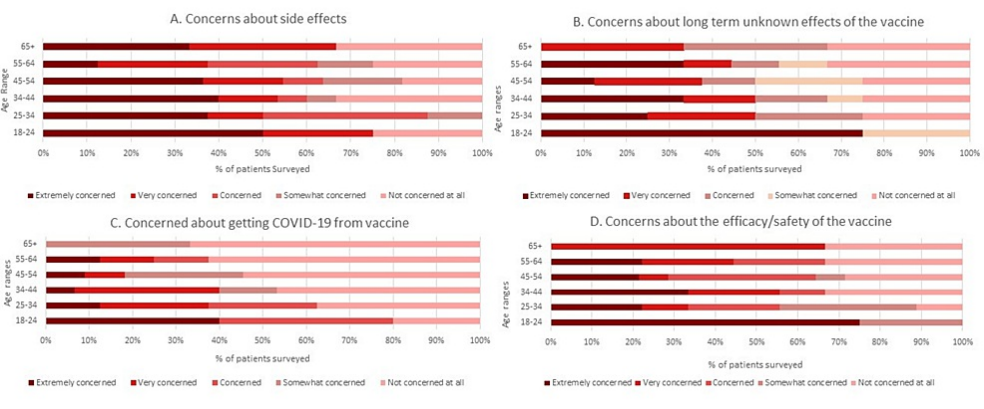
COVID-19 Vaccine Hesitancy Survey Results A: Concerns about side effects. B: Concerns about long-term unknown effects of the vaccine. C: Concerns about getting COVID-19 from vaccine. D: Concerns about the efficacy/safety of the vaccine.

Our project surveyed 53 individuals to assess their baseline concerns surrounding COVID-19 vaccination. When asked if patients have “concerns about side effects of the COVID-19 vaccine,” 60%-90% of participants reported being concerned to extremely concerned about this among the different age groups (Figure 2A). When asked specifically about the “long-term effects of the COVID-19 vaccine not being known,” 75% of the 18- to 24-year-old cohort reported being extremely concerned, with other age groups showing significant concern as well (Figure 2B). When asked “Are you worried about getting COVID-19 from the vaccine itself?,” 80% of individuals 18-24 years old reported being very concerned or extremely concerned (Figure 2C). When asked the question “Are you concerned about the efficacy and safety of the vaccine?,” up to 75% of the 18- to 24-year-old cohort once again reported being extremely concerned (Figure 2D).

Effects of COVID-19 vaccine education on patient perceptions

**Figure 3:**
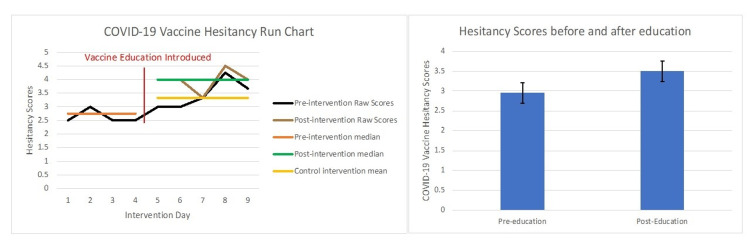
COVID-19 Vaccine Hesitancy Before and After Targeted Education Hesitancy scores: 0-5 (hesitant to not hesitant).

Vaccine hesitancy scores were calculated before and after targeted education. On average, we saw a mean increase in patients’ hesitancy scores from 3.3 to 4 out of 5 (Figure 3), with a higher score signifying a decrease in hesitancy. This increase was statistically significant (p < .01). This increase correlates to a change in patients' attitudes surrounding the vaccine, going from being more likely to delay getting the vaccine until more information is available to get it when they had free time.

Outcomes related to COVID-19 vaccine education

**Figure 4:**
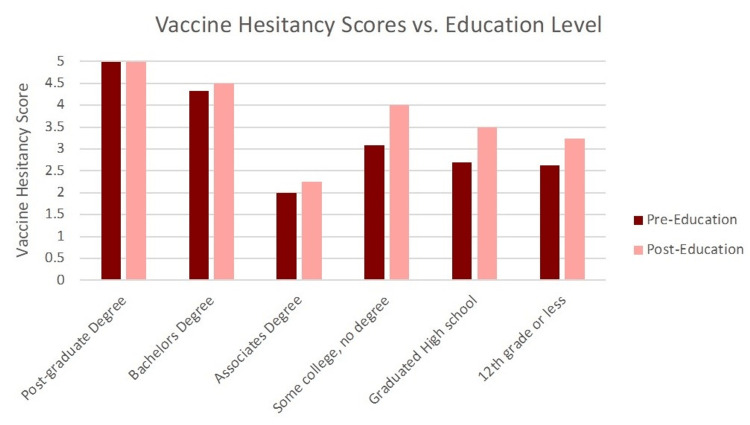
COVID-19 Vaccine Hesitancy Stratified by Education Level

**Figure 5:**
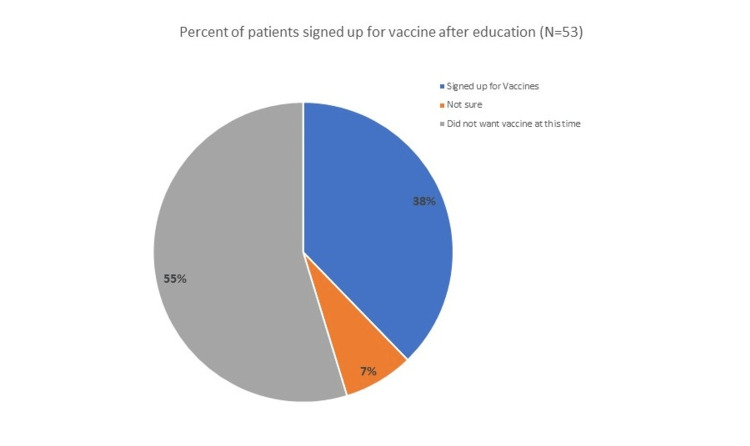
Percentage of Interviewed Patients Who Signed Up for COVID-19 Vaccine Appointments

Vaccine hesitancy scores were stratified by education level, and we found that apart from the associates' degree category (N = 4), an increase in educational attainment directly correlated with reduced vaccine hesitancy in our patient population (Figure 4). Additionally, of those who received targeted education, 38% agreed to sign up for a vaccine appointment at the conclusion of the survey, with 7% not sure but considering signing up (Figure 5).

## Discussion

ED visits represent an opportunity for public health education. With unlimited information readily available at our fingertips, it can be assumed that everyone is informed about the scientifically validated risks of medical interventions like vaccination. However, when taking a broad look at our ED administered vaccine hesitancy survey, we noted inconsistencies. Overall, it appeared that many individuals from our convenience sample had significant concerns surrounding the vaccines, sometimes regarding myths that were simply not based on science and data. One particularly surprising survey result was the consistent concern about getting COVID-19 from the vaccine itself. Eighty percent of the 18- to 24-year-old cohort said this played a significant role in their vaccine hesitancy. Thus, for the remainder of the vaccine education program, a concerted effort was made to dispel this notion specifically in our younger patients. This led to a significant improvement in vaccine acceptance. This is just one example of the many benefits that can be obtained by using surveys and targeted education based on the results of those surveys to lead public health education initiatives.

Our team initially decided to focus on vaccine hesitancy scores so that we could have a standardized method for assessing our patients' concerns surrounding COVID-19 vaccination. We were surprised to find that our patient population was resistant to new public health measures with baseline hesitancy scores averaging 2.75. A hesitancy score of around 3 correlates to patients saying they want to delay getting a COVID-19 vaccine and would not talk to a family member about their concerns or possibly getting the vaccine. Over the course of the pandemic, as public knowledge became more available, as well as friends and family getting a COVID-19 vaccine, hesitancy scores improved naturally as we expected. Around the time of our intervention, baseline hesitancy scores averaged 3.3. However, we found that after targeted vaccine education, hesitancy scores improved to 4 out of 5. This increase correlates to patients responding positively to the following two statements: 1) they will talk to family members about getting the vaccine, and 2) they are planning on getting the COVID-19 vaccine when they have time. While numerically these changes might seem insignificant, a change in patients' vaccine hesitancy from neutral to likely to get the vaccine cannot be understated. When expanded to the population at large, a decrease in vaccine hesitancy could lead to significantly improved vaccination rates and decreased mortality from COVID-19.

Coordinating a quality improvement project involving patient-centered vaccine education program in the ED setting was difficult; however, 38% of surveyed patients registered to receive a vaccine which demonstrates the potential impact and importance of ED-based public health education. These additional vaccines potentially prevented COVID-19-associated healthcare spending, morbidity, and mortality. Providing patients with evidence-based information, combined with shared decision making, is an incredibly effective method to improve vaccine compliance. Expanding public health education programs like this one could have an enormous impact on vaccine initiatives and preventative health outcomes in the future.

While these data capture a transient picture of this URM community’s hesitancy regarding the COVID-19 vaccine, the personal stories surrounding patients’ reasons for declining a vaccine were often moving. Multiple patients stated they could not trust any therapy coming from Johnson & Johnson (New Brunswick, USA) after reports of ovarian cancer were linked to talc powder sold by the company. When asked about the other vaccine companies like Pfizer (New York, USA) and Moderna (Cambridge, USA), patients often responded by saying that “all pharmaceutical companies are the same,” in a point-blank refusal. Most patients who came into the emergency department had their mindset. Some had trust in the healthcare system, had family members in healthcare, or had people close to them get the vaccine without major issues. Others stated that “our community does not get vaccines,” as they believe vaccines are unnatural and do not know what is in them. The speed at which the vaccine was rolled out and the polarizing political landscape surrounding both the pandemic and vaccine development also fueled concern among many patients. Many saw the government as playing a large role in pushing the vaccine approval process. With the current polarizing political landscape, patients’ mistrust in the government often extends into the health sector and was a significant factor in receiving the vaccine. Even after detailed education about many common concerns, it was often very difficult to impact patients’ vaccine-related decision making. However, education was effective for some patients, validating our attempt to educate all patients.

There were limitations to our quality improvement vaccine education program, and with some changes, future attempts may be more effective. One barrier to vaccination was having a supply of the vaccine on-site during a wide range of times. Ideally, patients would be offered vaccines immediately following the intervention. However, due to refrigeration requirements, vaccine scarcity, and staffing limitations, this was not possible. Signing patients up for COVID-19 vaccine appointments is an effective method for improving vaccine uptake; however, there is a risk that patients will not keep their vaccine appointments. Attempts were made to mitigate this barrier by ensuring that patients had transportation at the time of scheduling. An additional limitation was the rapidly changing political narrative of COVID-19 and vaccination. The release of Johnson & Johnson and AstraZeneca (Cambridge, England) blood clot concerns in early 2021 undoubtedly deterred many people from getting a vaccine. Almost every patient in the weeks following the news had concerns about these reports. To address these concerns, the intervention team had up-to-date information on the latest COVID-19 vaccine news and was able to answer questions accordingly. The impact of the blood clot news highlights the importance of rapid adaptation of the healthcare system to address public concerns before trust is lost permanently by some individuals. Finally, while this survey provided an intimate look into the reasons for COVID-19 vaccine hesitancy, it examined a small, specific audience of unvaccinated, eligible patients in a predominantly underserved urban hospital. Given the varied opinions across different populations and communities throughout the United States, a broader sample population is required to explore the additional reasons for COVID-19 vaccine hesitancy. To generalize these data to the entire population, a much larger sample with multiple sites would be required.

## Conclusions

The COVID-19 pandemic illustrated the enormous health disparities that exist within this country, particularly among URM and underserved communities. It also demonstrated the unique role that emergency departments have in providing care to underserved populations. The importance of the emergency department in shaping public health discourse and impacting patient perception cannot be overstated. Targeting efforts to provide education and support to these communities in the ED setting could play a role in reducing healthcare disparities relating to vaccination programs in the future.
